# Association between epicardial adipose tissue and myocardial work by non-invasive left ventricular pressure-strain loop in people with suspected metabolic syndrome

**DOI:** 10.1038/s41598-023-41779-x

**Published:** 2023-09-02

**Authors:** Li-juan Sun, Cheng-wei Xiao, Xue-bing Zhao, Shuai Guo, Fang Zhang

**Affiliations:** 1 Department of Ultrasound, First Hospital of Qinhuangdao, Qinhuangdao, 066000 Hebei China; 2 Hebei Medical University, Shijiazhuang, China

**Keywords:** Cardiology, Endocrinology, Medical research

## Abstract

Given the inconsistent results on the prognostic significance of epicardial adipose tissue (EAT), the purpose of the present study was to investigate the association of EAT thickness and myocardial work by non-invasive left ventricular pressure-strain loop in people with suspected metabolic syndrome (MS). A total of 194 participants imaged with echocardiography were evaluated. In accordance with the median EAT thickness, MS patients fell into thin EAT group and thick EAT group. Conventional echocardiographic parameters, global longitudinal strain (GLS) and the global myocardial work parameters obtained by pressure-strain loop analysis, comprising the global work index (GWI), global work efficiency (GWE), global constructive work (GCW) and global wasted work (GWW) were compared between the two groups. In comparison with the thin EAT group, thick EAT group achieved significantly higher values in interventricular septal thickness, end-diastolic left ventricular posterior wall thickness, left ventricular mass index and GWW (*p* < 0.05). while the absolute value of GLS, GWI, GCW, and GWE were notably lower in the thick EAT group (*p* < 0.001). EAT thickness showed a significant correlation with GWI and GCW (r = − 0.328, *p* = 0.001; r = − 0.253, *p* = 0.012), and also independently correlated with GWI and GCW in the multivariate regression analysis (β = − 0.310, *p* = 0.001; β = − 0.199, *p* = 0.049). EAT thickness is associated with left ventricular myocardial function in subjects with suspected metabolic syndrome, independently of other risk factors. Further studies are supposed to ensure the causal associations and related mechanisms.

## Introduction

Epicardial adipose tissue (EAT) refers to a type of visceral fat located between the myocardial surface and the visceral layer of the pericardium. EAT has been confirmed as an independent risk factor for accelerated progression of subclinical coronary atherosclerosis^1^; it is correlated with abdominal fat mass and obesity severity while showing an independent association with cardiovascular events^2^. EAT is considered to be an independent predictor of visceral obesity and may be closely associated with the pathogenesis of metabolic syndrome (MS)^3,4^, adverse clinical outcomes of COVID-19^5–7^, coronary artery disease, and diabetes^8^.

MS represents a group of common cardiovascular risk factors, comprising insulin resistance, obesity, atherosclerotic dyslipidemia, as well as hypertension^9^. Several cardiovascular risk factors occur together and are interrelated, with common potential mediators, mechanisms, and pathways^10^. MS turns out to be a leading health problem for its correlation with cardiovascular disease. It was reported to be correlated with a twofold increase in cardiovascular outcomes and a 1.5-fold increase in all-cause mortality^11^. It is clear why MS is the focus of various studies. The prevalence of MS has increased significantly worldwide, making accurate assessment of changes in left ventricular (LV) systolic function particularly important at an early stage. Tadic et al.^12^ evaluated left ventricular mechanics in patients with metabolic syndrome through two-dimensional echocardiography speckle tracking analysis, but the impact of metabolic syndrome on left ventricular myocardial work has not yet been evaluated.

Ejection fraction (EF) is the most commonly used index of LV systolic function examined by conventional echocardiography. However, the measurement of EF is a simplistic method, limited by the requirements of geometric assumptions. Thus, in high-risk subclinical patients with MS, subtle changes may be overlooked when myocardial systolic function is assessed using EF alone. The use of two-dimensional speckle tracking echocardiography for myocardial deformation imaging has made the progress of left ventricular quantification beyond EF and become an excellent prognostic marker of cardiac events. However, one of the main limitations of strain imaging is load dependence, which may affect the diagnostic accuracy of myocardial function assessment. LV pressure-strain loop (PSL) analysis refers to a novel echocardiographic method for noninvasive quantification of myocardial work^13^. Based on the speckle tracking echocardiography, brachial artery cuff pressure has been employed to replace left ventricular pressure, such that the LV strain and pressure parameters are combined, which can reduce the influence of afterload on the myocardial strain. Currently, limited data are available regarding the changes in left ventricular myocardium work and the correlation between epicardial fat and left ventricular myocardium work in MS patients.

Based on the above, we hypothesized that the accumulation of EAT in patients with metabolic syndrome is related to sub-clinical changes in left ventricular myocardial work. Therefore, in this study, we aimed to investigate left ventricular systolic function and myocardial work in patients with suspected metabolic syndrome using left ventricular pressure-strain loop technique. Furthermore, we assessed the correlation between EAT thickness and left ventricular myocardial work in patients with suspected metabolic syndrome.

## Methods

### Study population

Among 213 suspected MS patients imaged with echocardiography from September 2020 to December 2021, 194 patients were recruited. The exclusion criteria for the 19 patients comprised poor image quality (n = 12), and arrhythmia (n = 7). The median patient age was 62 years, 95 patients (49%) were men. The MS was defined based on the presence of three or more components of the National Cholesterol Education Program Adult Treatment Panel III (NCEP-ATP III) criteria (2005 Revision)^14^: Elevated triglycerides > 1.7 mmol/L; reduced high‐density lipoprotein cholesterol (HDL-C) (< 1.3 mmol/L in women and < 1.03 mmol/L in men); hypertension (HTN) (≥ 130/85 mmHg); hyperglycaemia ≥ 100 mg/dL; abdominal obesity by waist circumference ≥ 80 cm in women and ≥ 90 cm in men^15^. Furthermore, the exclusion criteria included participants with a previous history of coronary artery disease, congenital heart disease, heart failure, atrial fibrillation, moderate or severe valvular heart disease, stroke, or any revascularization. This study protocol gained approval from the Ethics Committee of the First Hospital of Qinhuangdao (2021Q082). The median EAT thickness reached 3.82 (Inter- Quartile Range 2.28) mm. In accordance with the median EAT thickness, MS patients were divided into two groups: thin EAT group (< 3.82 mm, n = 97) and thick EAT group (≥ 3.82 mm, n = 97). The clinical data, laboratory examination, echocardiography and myocardial work were compared between the two groups.

### Clinical and laboratory data

Anthropometry (height, weight) was performed for all participants in the study to calculate body mass index (BMI). Moreover, waist circumference, heart rate, systolic (SBP) and diastolic blood pressures (DBP) were examined. Fasting plasma glucose (FPG), uric acid, triglyceride (TG), total cholesterol (TC), high-density lipoprotein cholesterol (HDL-C), and low-density lipoprotein cholesterol (LDL-C) were examined less than two weeks prior to the echocardiographic evaluation using standard laboratory techniques.

### Echocardiographic analysis

Echocardiographic examinations were performed using Vivid E95 (GE Vingmed, Horten, Norway) ultrasound machine equipped with a M5S 3.5 MHz transducer (frame rate 50 ~ 80 frames/s). All records and measurements were taken according to American Association of Echocardiography guidelines^16^. From the parasternal long axis view, we examined ascending aorta diameter (AAO), left atrial diameter (LAD), LV end-diastolic diameter (LVEDd), end-diastolic LV posterior wall (LVPWd) and interventricular septal (IVSd) thickness. LV ejection fraction (LVEF) was calculated using the modified Simpson's rule from apical 4- and 2-chamber views. LV mass (LVM) was determined using the Devereux formula^17^: LVM (g) = 0.8 × 1.04 × [(LVDd + IVS + LVPW)^3^ − LVDd^3^] + 0.6. The LV mass index (LVMI) was calculated by dividing the LVM by body surface area (BSA). BSA was calculated according to the Dubois formula^18^: BSA = 0.007184*Height^0.725^*Weight^0.425^. Pulse wave Doppler was adopted to measure the peak velocity of early and late mitral diastolic blood flow (E and A), tissue Doppler imaging was employed to measure the early diastolic motion velocity of mitral ring (e'), and E/e' were obtained.

### EAT thickness measurement

EAT refers to the fat pool between the outer wall of the myocardium and the visceral layer of the pericardium. The thickness of EAT was measured by echocardiography. Maximum EAT thickness was examined perpendicular to right ventricular free wall at the end of systole in a parasternal long-axis view. Three different measurements were recorded over three consecutive cardiac cycles, and the mean value of EAT was determined.

### LV strain and myocardial work quantification

GE EchoPAC version 203 software was employed for LV strain and myocardial work analysis. Three apical (i.e., long-axis, four and two-chamber) views were captured and then stored for subsequent off-line analysis (average 3 consecutive cycles). Aortic valve closure was automatically recognized in apical long axis view. Click automated function imaging (AFI) button, the system automatically recognized the apical 4-chamber and 2-chamber view, and tracked the contour of the left ventricle endocardial and ventricular wall. The areas of interest were regulated by correcting endocardium boundaries or widths if required. The global longitudinal strain (GLS) was determined based on the weighted average of the peak systolic longitudinal strains at all 17 segments. After GLS was obtained, insertion brachial artery blood pressure, and timing of valve events, the software derived a non-invasive PSL. The global myocardial work parameters (e.g., the global work index (GWI), global work efficiency (GWE), global constructive work (GCW), and global wasted work (GWW)) were obtained through the PSL analysis.

### Statistical analysis

The data were analyzed using SPSS software (version 27). Continuous variables are expressed as mean ± standard deviation if normally distributed or median with interquartile ranges if not normally distributed. Data are expressed as percentages in terms of categorical variables. Statistical comparisons comprised student t-test or Mann–Whitney U test. The correlation between different metabolic syndrome criteria and LV myocardial work parameters was determined using Pearson correlation coefficient. The variables with *p* values ≤ 0.10 were included into the stepwise multiple regression analysis. A total of 20 patients were randomly selected and examined by two experienced cardiac sonographers. The consistency analysis of GWI, GCW, GWW and GWE was performed, and the same samples were analyzed again by one of the radiologists one week later. Intra-observer and inter-observer variables were calculated by intra-class correlation coefficient (ICC). A *p* value less than 0.05 was considered a difference that achieved statistical significance.

### Ethics approval and consent to participate

Informed consent was obtained from all subjects and/or their legal guardian(s). All methods were carried out in accordance with relevant guidelines and regulations. This study was approved by the Ethics Committee of the First Hospital of Qinhuangdao (2021Q082).

## Results

### Study population characteristics

The baseline characteristics of MS patients are shown in Table 1. MS patients in the thick EAT group were characterized by higher BMI, SBP, DBP, waist circumference and triglyceride than patients in the thin EAT group (26.89(4.11) vs. 27.73(5.25), *p* < 0.05; 138(10) vs. 140(20), *p* < 0.05; 80(12) vs. 85(12.25), *p* < 0.05; 93(12) vs. 97(15.75), *p* < 0.05; 2.01(1.08) vs. 2.59(2.74), *p* < 0.01). No significant differences were identified between the two groups in age, male proportion, heart rate, fasting blood glucose, uric acid, total cholesterol, LDL-C and HDL-C (*p* > 0.05).

**Table 1:** Patient characteristics of this study.

Characteristics	Thin EAT (n = 97)	Thick EAT (n = 97)	*p* value
Age (years)	63 (11)	62 (18)	NS
Male, n (%)	42 (43.30)	53 (54.64)	NS
BMI (kg/m^2^)	26.89 (4.11)	27.73 (5.25)	0.016
Heart rate (beat/min)	73 (16.50)	77 (16.75)	NS
SBP (mm Hg)	138 (10)	140 (20)	0.013
DBP (mm Hg)	80 (12)	85 (12.25)	0.021
Waist circumference (cm)	93 (12)	97 (15.75)	0.029
Fasting blood glucose (mg/dl)	7 (3.17)	7.5 (3.07)	NS
Uric acid (μmol/l)	346.73 ± 95.93	366.22 ± 119.67	NS
Triglyceride (mmol/l)	2.01 (1.08)	2.59 (2.74)	0.002
Total cholesterol (mmol/l)	5.10 ± 1.43	5.03 ± 1.34	NS
LDL-C (mmol/l)	2.80 ± 0.94	2.83 ± 0.89	NS
HDL-C (mmol/l)	1.07 (0.42)	1.12 (0.49)	NS

*EAT* epicardial adipose tissue, *BMI* body mass index, *SBP* systolic blood pressure, *DBP* diastolic blood pressure, *LDL-C* high-density lipoprotein cholesterol, *HDL-C* low-density lipoprotein cholesterol, *NS* not significant.

### Comparison of conventional echocardiographic parameters and GLS

Table 2 lists the conventional echocardiographic parameters and GLS. No significant difference was reported between the thin and thick EAT groups in AAO, LAD, LVEDd, LVEF, A and E/e′ (*p* > 0.05). However, in comparison with the thin EAT group, thick EAT group showed significantly higher values in IVSd, LVPWd and LVMI (10(3) vs. 11(4), *p* < 0.05; 8(1) vs. 9(2), *p* < 0.001; 79.24(25.95) vs. 85.97(32.22), *p* < 0.05). The absolute value of GLS, E and e′ were significantly lower in the thick EAT group (17(2) vs.14(2.75), *p* < 0.001; 69.61(19.50) vs. 64(20.22), *p* < 0.05; 6.6(1.85) vs. 6(2.20), *p* < 0.01).

**Table 2:** Comparisons of conventional echocardiographic parameters and GLS between thin and thick EAT groups.

Characteristics	Thin EAT (n = 97)	Thick EAT (n = 97)	*p* Value
AAO (mm)	34 (4)	34 (5)	NS
LAD (mm)	36 (4)	37 (4)	NS
IVSd (mm)	10 (3)	11 (4)	0.035
LVPWd (mm)	8 (1)	9 (2)	< 0.001
LVMI (g/m^2^)	79.24 (25.95)	85.97 (32.22)	0.018
LVEDd (mm)	47 (4)	48 (5.75)	NS
LVEF (%)	65 (4)	65 (4)	NS
E (cm/s)	69.61 (19.50)	64 (20.22)	0.033
A (cm/s)	91.07 ± 19.69	88.69 ± 19.44	NS
e′ (cm/s)	6.6 (1.85)	6 (2.20)	0.006
E/e′	10.73 (3.69)	10.96 (3.97)	NS
GLS (%)	− 17 (2)	− 14 (2.75)	0.000

*EAT* epicardial adipose tissue, *AAO* ascending aorta diameter, *LAD* left atrial diameter, *IVSd* interventricular septal thickness, *LVPWd* end-diastolic left ventricular posterior wall thickness, *LVMI* left ventricular mass index, *LVEDd* left ventricular end-diastolic diameter, *LVEF* left ventricular ejection fraction, *E* the peak velocity of early mitral diastolic blood flow, *A* the peak velocity of late mitral diastolic blood flow, *GLS* global longitudinal strain, *e'* early diastolic motion velocity of mitral ring, *NS* not significant.

### Left ventricular myocardial work

**Figure 1:**
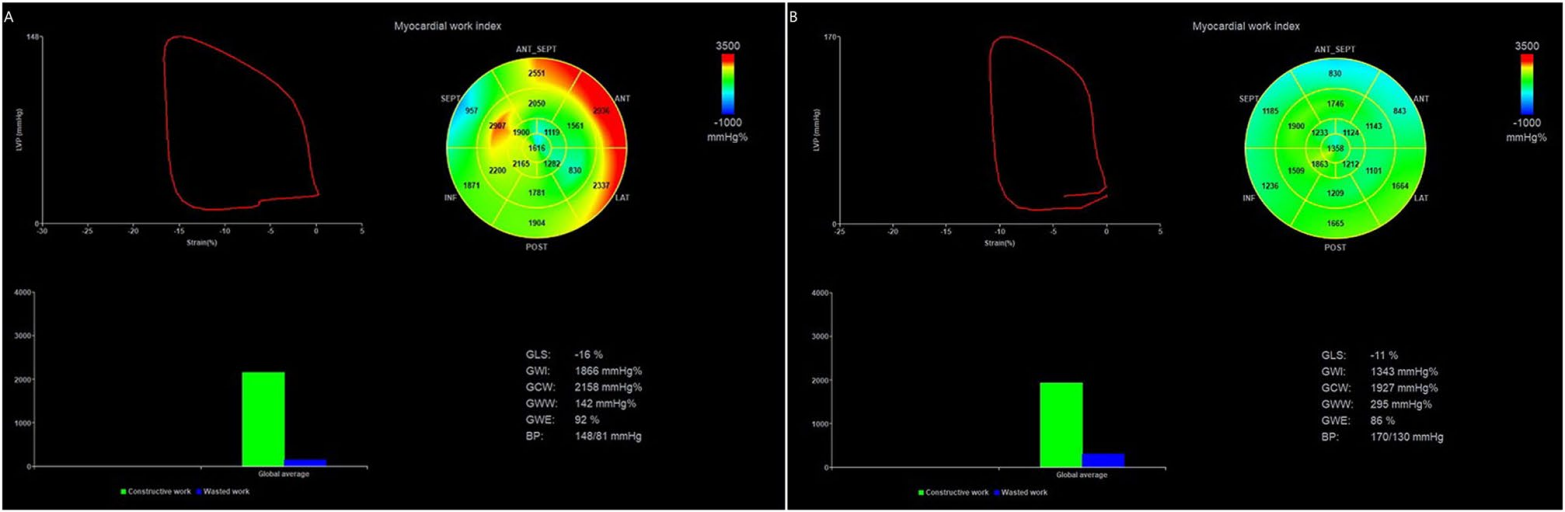
Representative cases of global work index by pressure-strain loop in thin (**A**) and thick (**B**) EAT groups. (**A**) GLS: − 16%; GWI:1866 mmHg%; GCW: 2158 mmHg%; GWW: 142 mmHg%; GWE: 92%; BP: 148/81 mmHg. (**B**) GLS: − 11%; GWI:1343 mmHg%; GCW: 1927 mmHg%; GWW: 295 mmHg%; GWE: 86%; BP: 170/130 mmHg. *EAT* epicardial adipose tissue, *GLS* global longitudinal strain, *GWI* global work index, *GCW* global constructive work, *GWW* global wasted work, *GWE* global work efficiency.

We further examined whether left ventricular myocardial work was affected by the change of EAT thickness. Detailed results on LV myocardial work are summarized in Table 3. Using the novel PSL analysis method, the thick EAT group achieved, in comparison with the thin EAT group, significantly higher value in GWW (105(85.5) vs.150(95.5); *p* < 0.001). The GWI (1817.58 ± 305.57 vs. 1497.21 ± 298.71; *p* < 0.001), GCW (2198.19 ± 337.61 vs. 1878.35 ± 310.95; *p* < 0.001), and GWE (94(3) vs. 91(5); *p* < 0.001) were significantly decreased in the thick EAT group (all *P* < 0.05). Figure 1 shows representative cases of global work index by pressure-strain loop in thin and thick EAT groups.

**Table 3:** LV global myocardia work assessment in the study population.

	Thin EAT (n = 97)	Thick EAT (n = 97)	*p* value
GWI (mmHg%)	1817.58 ± 305.57	1497.21 ± 298.71	< 0.001
GCW (mmHg%)	2198.19 ± 337.61	1878.35 ± 310.95	< 0.001
GWW (mmHg%)	105 (85.5)	150 (95.5)	< 0.001
GWE (%)	94 (3)	91 (5)	< 0.001

*EAT* epicardial adipose tissue, *GWI* global work index, *GWE* global work efficiency, *GCW* global constructive work, *GWW* global wasted work.

### Correlation and multivariate regression of metabolic syndrome risk factors and left ventricular myocardial work

**Figure 2:**
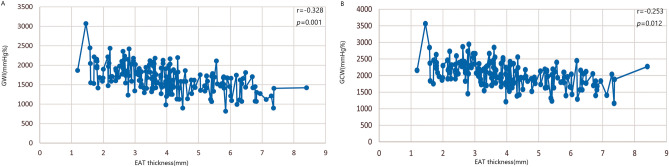
The correlation of EAT thickness and left ventricular myocardial work. There was a negative correlation between EAT and GWI (**A**), GCW (**B**) (r_1_ = − 0.328, r_2_ = − 0.253, *p* < 0.05). *EAT* epicardial adipose tissue, *GWI* global work index, *GCW* global constructive work.

GWI and GCW were significantly correlated with EAT thickness (r = − 0.328, *p* = 0.001; r = − 0.253, *p* = 0.012) (Fig. 2, Table 4) and SBP (r = 0.542, *p* < 0.001; r = 0.524, *p* < 0.001). Moreover, GCW showed a weak correlation with triglyceride (r = − 0.200, *p* = 0.049). GWW and GWE had notable correlations with fasting blood glucose (r = 0.279, *p* = 0.006; r = − 0.217, *p* = 0.033) (Table 4).

**Table 4:** The correlation and multivariate regression of the metabolic syndrome risk factors and left ventricular myocardial work.

	GWI (mmHg%)		GCW (mmHg%)		GWW (mmHg%)		GWE (%)	
	Correlation *r*	Multivariate regression β	Correlation *r*	Multivariate regression β	Correlation *r*	Multivariate regression β	Correlation *r*	Multivariate regression β
EAT	− 0.328**	− 0.310**	− 0.253*	− 0199*	0.039		− 0.117	
Age	0.012		0.088		0.145		− 0.139	
BMI	0.043		− 0.010		0.041		− 0.055	
SBP	0.542**	0.478**	0.524**	0.451**	0.066		− 0.016	
DBP	0.041		0.078		0.083		− 0.028	
Fasting blood glucose	− 0.019		0.130		0.279**	0.248*	− 0.217*	− 0.195
Triglyceride	− 0.126		− 0.200*	− 0.090	− 0.179		0.176	
Total cholesterol	0.063		0.025		− 0.040		0.057	
HDL-C	0.101		0.062		− 0.101		0.108	
LDL-C	0.153		0.177		0.070		− 0.038	
*R*^2^		0.445		0.326		0.184		0.120

*EAT* epicardial adipose tissue, *BMI* body mass index, *SBP* systolic blood pressure, *DBP* diastolic blood pressure, *HDL-C* low-density lipoprotein cholesterol, *LDL-C* high-density lipoprotein cholesterol, *GWI* global work index, *GCW* global constructive work, *GWW* global wasted work, *GWE* global work efficiency.

*P < 0.05, **P < 0.01.

As indicated by the result of multivariate regression analysis, GWI and GCW were independently correlated with EAT thickness (β = − 0.310, *p* = 0.001; β = − 0.199, *p* = 0.049) and SBP (β = − 0.478, *p* = 0.000; β = − 0.451, *p* < 0.001). GWW was independently correlated with fasting blood glucose (β = 0.248, *p* = 0.011) (Table 4).

### Intra- and inter-observer variability

Table 5 lists the myocardial work parameters for the 20 subjects. The intra-observer variability showed nearly perfect agreement for GWI, GCW, GWW, and GWE. The inter-observer variability of GWI, GCW, GWW, and GWE also showed very strong correlations.

**Table 5:** Inter-class correlation coefficient for intra- and inter-observer variability for myocardial work parameters.

	Intra-observer variability			Inter-observer variability		
	ICC	95% CI	*p* value	ICC	95% CI	*p* value
GWI (mmHg%)	0.978	0.945 ~ 0.991	< 0.001	0.929	0.830 ~ 0.971	< 0.001
GCW (mmHg%)	0.949	0.875 ~ 0.979	< 0.001	0.898	0.762 ~ 0.959	< 0.001
GWW (mmHg%)	0.998	0.996 ~ 0.999	< 0.001	0.972	0.931 ~ 0.989	< 0.001
GWE (%)	0.936	0.846 ~ 0.974	< 0.001	0.815	0.590 ~ 0.922	< 0.001

*GWI* global work index, *GCW* global constructive work, *GWW* global wasted work, *GWE* global work efficiency, *ICC* inter-class correlation coefficient, *CI* confidence interval.

## Discussion

This is the first study to manifest the correlation between EAT thickness and LV myocardial work in subjects with suspected metabolic syndrome. The main findings of the study were: (1) Compared with the thin EAT group, IVSd, LVPWd and LVMI in the thick EAT group were significantly increased, while the absolute values of GLS, E and e' were significantly decreased; (2) The thick EAT group achieved significantly higher value in GWW in comparison with the thin EAT group. Meanwhile, the GWI, GCW, and GWE were significantly decreased in the thick EAT group; and (3) EAT thickness was inversely correlated with GWI and GCW.

### EAT and left ventricular remodeling

In general, the conventional clinical evaluation of left ventricular systolic function is performed through the measurement of myocardial fiber shortening indexes (e.g., left ventricular EF, left ventricular wall thickening, myocardial velocity, and strain)^19^. In this study, conventional echocardiographic parameters were compared between the two groups. The results show that IVSd, LVPWd and LVMI in the thick EAT group were significantly increased compared with the thin EAT group, while the absolute values of GLS, E and e' were significantly decreased. In metabolic syndromes, left ventricular hypertrophy may occur with increased after-load^20^. It is the most prominent early cardiac manifestation in hypertensive patients, characterized by increased myocardial weight and ventricular remodeling. Diabetic myocardial microangiopathy can lead to chronic myocardial ischemia and hypoxia, cardiomyocyte necrosis, myocardial fibrocyte proliferation, and eventually left ventricular hypertrophy. Moreover, several inflammatory substances released from adipocytes, such as resistin have been suggested to contribute toward the adverse effects of obesity on the heart by promoting myocardial hypertrophy and dysfunction^21^. Extensive research has reported that the myocardial strain obtained by two-dimensional speckle tracking imaging can make left ventricular quantization beyond LVEF a better prognostic marker of cardiac events^22–24^. In this study, no significant difference in AAO, LAD, LVEDd, LVEF, A and E/e′ was reported between the thin and thick EAT groups. Compared with the traditional index of systolic function, GLS is more sensitive to indicate the degree of myocardial injury, and may decrease prior to the change of LVEF, consistent with previous literature reports. Among the measurements of myocardial function, GLS measured by echocardiography is the most widely studied marker, providing a simple, inexpensive, and quantitative way to assess global long-axis systolic function. However, one of the major limitations of strain imaging is after-load dependence. Moreover, it cannot reflect myocardial work or oxygen demand^25^. The noninvasive LV pressure-strain loop serves as a novel method of quantifying myocardial work in combination with myocardial strain and LV pressure measurements, and may provide incremental value for assessment of myocardial function^26^. The work contributed by the respective normal contracting component is positive and is called "constructive work". In the normal heart, very little work is wasted and thus there is a high work efficiency. Compared with the thin EAT group, GWW was significantly increased in thick EAT group, while GWI, GCW, and GWE notably declined. Prolonged systole and shortened myocardium after aortic valve closure will produce wasted work because it does not result in left ventricular ejection. In early untreated hypertension, diastolic dyssynchrony mainly affected constructive work, whereas post systolic shortening affected wasted work^27^.

### EAT and left ventricular myocardial work

To the best of our knowledge, this study has been the first to evaluate the role of MS on the correlation between EAT and myocardial work. The EAT is located outside the myocardial wall and maintains intimal contact with the epicardial vessels and myocardium, allowing for paracrine or vasocrine effects. Under health conditions, EAT has protective functions (e.g., prevention of hypothermia, secretion of adiponectin by epicardial fat cells, and mechanical protection of coronary circulation) while taking on critical significance in the energy supply of myocardium^28^. However, excess body fat accumulation is correlated with metabolic abnormalities and myocardial dysfunction^29,30^. In this case, the protective properties of EAT may be destroyed and become harmful tissues that promote the development of cardiovascular diseases. Despite the unclear underlying mechanism, EAT has been confirmed as a potential source of inflammatory mediators, comprising interleukin (IL)-1β, IL-6, and tumor necrosis factor (TNF)-α. Hirata et al. reported that the infiltration of inflammatory cells was enhanced in the epicardial adipose tissue of patients with coronary artery disease, instead of not in the subcutaneous fat^31^. Moreover, EAT has the potential to secrete adipotropic factors and free fatty acids, which may promote myocardial steatosis and induce cellular oxidative stress, as well as increased nitric oxide synthase activity and production of intracellular nitric oxide, and eventually lead to apoptosis of myofibrillar cells^32^. Recent research has placed a focus on myocardial fibrosis, a critical histological component of cardiac remodeling. Myocardial fibrosis scarring occurs most commonly after myocardial infarction, but can also occur in a variety of other diseases that promote myocardial fibrosis (e.g., hypertensive heart disease, diabetic hypertrophic cardiomyopathy, and idiopathic dilated cardiomyopathy)^33^. As revealed by relevant data, EAT is capable of producing and secreting adipo-fibrokines (e.g., Activin A), which may promote the formation of myocardial fibrosis^34^.

A significant correlation and agreement existed between the area of the non-invasive LV pressure-strain loop and glucose metabolism examined by positron emission tomography. Glucose metabolism is capable of indicating myocardial work. GCW refers to the work performed by the LV during contraction that contributes to LV ejection and is divided into shortening of cardiomyocytes during contraction and lengthening of cardiomyocytes during isovolumic relaxation. GWI is the total work in the LV pressure-strain loop area calculated from mitral valve closure to mitral valve opening^26^. The left ventricular mechanics were significantly impaired by speckle tracking echocardiography in patients with metabolic syndrome. Previous studies have shown that epicardial fat volume is negatively correlated with left ventricular systolic dysfunction in patients with heart failure^35^. In this study, we found that EAT was negatively correlated with GWI and GCW in myocardial work parameters in patients with metabolic syndrome. In middle-aged male subjects with metabolic syndrome, EAT was correlated with inflammation represented by high‑sensitivity C‑reactive protein level, LV mass index, e′ and GLS, suggesting that the inflammatory activity of EAT induced myocardial remodeling and dysfunction^29^. Besides, this study further confirmed that EAT can have an adverse relationship with LV myocardial work at the early stage of LV remodeling.

### LV myocardial work and metabolic syndrome criteria

Furthermore, LV myocardial work were also correlated with SBP, triglyceride, fasting blood glucose in metabolic syndrome. Men and women with impaired fasting glucose and impaired glucose tolerance have more severe alterations in cardiometabolic profiles and inflammatory markers than patients with impaired fasting glucose alone. Even in the absence of high blood pressure, these people were ten times more likely to develop preclinical cardiovascular disease^36^. Fibroblast growth factor-23 (FGF-23) is a biomarker of cardiovascular disease and may also be an early marker of cardiac injury in obese but otherwise healthy African American adolescents. Obesity may promote FGF-23 production in the absence of chronic kidney disease^37^. Previous studies have shown that blood pressure, waist circumference, and fasting glucose levels exert the most significant effect on left ventricular deformation of all metabolic syndrome components^38^. The findings in this study are similar to those of previous studies.

### Limitations

Several limitations remained in this study. The correlation between increased EAT thickness and impaired LV myocardial work cannot prove that increased EAT inevitably leads to increased LV injury, and its pathophysiological mechanism needs to be further demonstrated. LV pressure-strain loop technology is based on two-dimensional speckle tracking echocardiography, which requires high-quality ultrasonic images. Brachial systolic pressure examined with a cuff and sphygmomanometer may be inaccurate because systolic pressure varies.

## Conclusion

Although no etiological relationships can be established from the current study, our intriguing finding of the independent relationship of EAT thickness and left ventricular myocardial function by LV pressure-strain loop in subjects with suspected metabolic syndrome may have important clinical implications. Moreover, EAT may serve as a useful marker for identifying metabolic syndrome patients at high risk of abnormal myocardial work. Nevertheless, there is no further mechanistic studies regarding associations between them. Further studies are supposed to ensure the causal associations and related mechanisms.

## Data Availability

Correspondence and requests for materials should be addressed to F.Z.
